# Factors Affecting Reading Speed in Patients with Diabetic Macular Edema Treated with Laser Photocoagulation

**DOI:** 10.1371/journal.pone.0105696

**Published:** 2014-09-29

**Authors:** Elizabeth Pearce, Sobha Sivaprasad, Ngaihang V. Chong

**Affiliations:** 1 Department of Ophthalmology, King's College Hospital, London, United Kingdom; 2 Department of Medical Retina, National Institute of Health Research Moorfields Biomedical Research Centre, London, United Kingdom; 3 Oxford Eye Hospital, Oxford University Hospitals, Oxford, United Kingdom; University of Utah (Salt Lake City), United States of America

## Abstract

**Purpose:**

To study the factors that may affect reading speed in patients with diabetic macular edema previously treated with laser photocoagulation.

**Methods:**

Consecutive patients with type II diabetes treated with laser photocoagulation for diabetic macular edema (DME) at least twelve months previously, with best corrected visual acuity of better than 65 letters (approximately 20/40) measured with Early Treatment Diabetic Retinopathy Study (ETDRS) charts were included in this study. Patients previously treated with pan-retinal photocoagulation, vitrectomy, intravitreal steroid or anti-VEGF therapy were excluded. Any other ocular co-morbidities that may influence reading ability such as cataract, glaucoma or macular degeneration were also excluded. All patients were refracted by a certified examiner, the following measurements were collected: best corrected visual acuity (BCVA), contrast sensitivity with Pelli-Robson chart, reading speed with MNREAD chart, microperimetry with Nidek MP1, and central subfield thickness with Zeiss spectral domain optical coherent topography.

**Results:**

The slow reading group had poorer contrast sensitivity (p = 0.001), reduced retinal sensitivity (p = 0.027) and less stable fixation (p = 0.013). Most interestingly the reduced retinal sensitivity findings were driven by the microperimetry value on the right subfield (p = 0.033), (nasal to the fovea in the right eye and temporal to the fovea in the left eye). Multiple linear regression analysis showed that contrast sensitivity is probably the most important factor that affects reading speed (p = 0.001).

**Conclusion:**

Reduced retinal sensitivity after laser treatment is associated with reduced reading speed in patients with diabetic macular edema.

## Introduction

Laser photocoagulation remains the first-line treatment for diabetic macular edema (DME) in most patients globally despite the availability of intravitreal steroids and inhibitors of vascular endothelial growth factor (VEGF). Recent clinical trials show that the visual acuity (VA) outcome of laser photocoagulation for DME is more favourable than the results of the Early Treatment Diabetic Retinopathy Study (ETDRS) [1]–[3]. Despite this, at an individual level, patients are often unhappy with their quality of vision after macular laser photocoagulation despite good recorded distance VA. This particularly applies to reading vision suggesting a discrepancy between distance and reading VA as seen in other macular diseases such as age-related macular degeneration (AMD) and uveitic macular edema [4]. Reading ability is a critical parameter for assessing the quality of life and the influence on the ability to perform everyday tasks. In contrast to AMD, DME predominantly affects the working age group and thus the potential socio-economic impact of poor quality of vision is more significant. In this study, we assessed various factors that may influence reading vision in patients with DME treated with macular laser to better understand the discrepancy between distance and reading vision.

## Methods

The study was performed in the Laser and Retinal Research Unit at King's College Hospital. The research adhered to the tenets of the Helsinki agreement, all patients gave informed consent to the study and the study was approved by the Chair of the Local Ethics Committee at King's College Hospital.

### Patients

Consecutive patients with type II diabetes who were treated with macular laser photocoagulation using the modified ETDRS grid treatment without treating the foveal avascularised zone for DME at least twelve months before enrolment with best corrected visual acuity of better than 65 letters (approximately 20/40) measured with Early Treatment Diabetic Retinopathy Study (ETDRS) charts were included in this study. The patients had good reading ability with English as their first language and no past history of dyslexia.

All patients with previously treated with pan-retinal photocoagulation, vitrectomy, intravitreal steroid or anti-VEGF therapy were excluded. Any other ocular co-morbidity that might influence reading ability such as cataract, glaucoma or macular degeneration was also excluded. Only eyes which met the study criteria were included in the study. All measurements and analysis were based on monocular examination.

### Visual acuity measurement

All patients were refracted by a certified examiner and best corrected visual acuity (BCVA) for each eye was measured using standard ETDRS protocol at 4 metres distance with a modified ETDRS distance chart. Visual acuity was scored as the total number of ETDRS letters read correctly, then converted to LogMar vision.

### Contrast sensitivity

Contrast sensitivity measurement was performed after visual acuity measurements, with the Pelli-Robson chart (Clement Clarke Inc., Harlow, UK) at a distance of 1 m and chart luminance of 80 to 120 cd/m^2^ measured with a luminance metre (Minolta Konics LS 110). The right eye was tested, followed by the left eye, on charts 1 and 2, respectively, with +0.75 DS added to the patient's refraction. The patient was asked to name each letter on the chart starting with the high-contrast letters in the top left-hand corner then reading horizontally across the entire line. The test was stopped when the patient failed to identify 2 or more letters correctly in a triplet.

### Reading Performance Measurement

After refraction, reading performance was measured by the certified examiner using a standardized protocol with the MNREAD acuity charts (Optelec, US). These contain 19 sentences of different print sizes ranging from +1.3 to −0.5 logMAR with each sentence containing 60 characters. The test was performed monocularly, the right eye tested first followed by the left eye, using charts with different sentences with a reading correction added to the patient's refraction to optimize reading at 40 cm. Chart luminance was 120 cd/m^2^.

The patients read aloud one sentence at a time as quickly and accurately as possible, as the sentences were uncovered one by one from large to small print. Reading time was recorded in seconds for each sentence using a stopwatch. The number of errors made for each sentence were recorded on a score sheet and converted to reading speed in words per minute by the method described in the test instructions.

The reading parameters were then calculated as follows:

The reading acuity (RA) in logMAR was calculated using the formula:

RA = 1.4 – (sentences read × 0.1) + (errors × 0.01)

Reading speed was calculated in words per minute. We used the formula:

Reading speed  = 60× (10 – errors)/(time in seconds)

The maximum reading speed (MRS) is the maximum speed recorded for any particular sentence during the test. Critical print size (CPS) is the smallest size sentence which can be read at or faster than 90% of the average of three fastest speeds recorded.

### Assessment of location and stability of fixation using MP-1 microperimetry and calculation of bivariate contour ellipse area (BCEA)

The Nidek microperimeter (MP1, Nidek Instruments, Italy) was used to measure fixation with a red fixation cross of height 2° presented on a dark background on the LCD screen of the microperimeter on the right eye after the other eye was occluded. Once patients had located the cross, fixation was measured for a period of 30 seconds. The eye position was recorded by tracking a retinal landmark at 25 Hz throughout the fixation assessment.

The pattern of fixation was classified based on location and stability using the MP-1 software.

Crossland et al [5] reported that quantifying fixation stability by calculating a bivariate contour ellipse area (BCEA) that encompasses 68% of fixations is a more powerful and accurate tool than the in-built fixation software of MP-1 for patients with macular diseases. The log BCEA correlated well with reading speed in patients with AMD. Fixation data was collected by the microperimeter (exported as a.mfd text file) over 30 seconds. The BCEA was calculated using Microsoft Excel (Microsoft Corporation, Seattle, WA, USA) based on the original formula published previously and the log BCEA of each study eye was recorded. As this method is more reliable, we have included this in the statistical analysis.

### Retinal sensitivity

The sensitivity of the central visual field was tested with a customised 14 points program using “white” test lights (stimulus size Goldmann III, duration 200 msec) presented on a dim “white” background (1.27 cd/m^2^) using a 4 –2 threshold strategy. The 4 – 2 threshold strategy starts with the initial attenuation value, the value is then decremented by 4 db at a time until the patient is capable of recognizing the stimulus. Once the intensity at which the stimulus is seen has been determined, it rises in 2 db steps until the patient is no longer able to detect the stimulus, then be decremented by 2 db at a time to determine the sensitivity threshold for the current stimulus.

**Figure 1:**
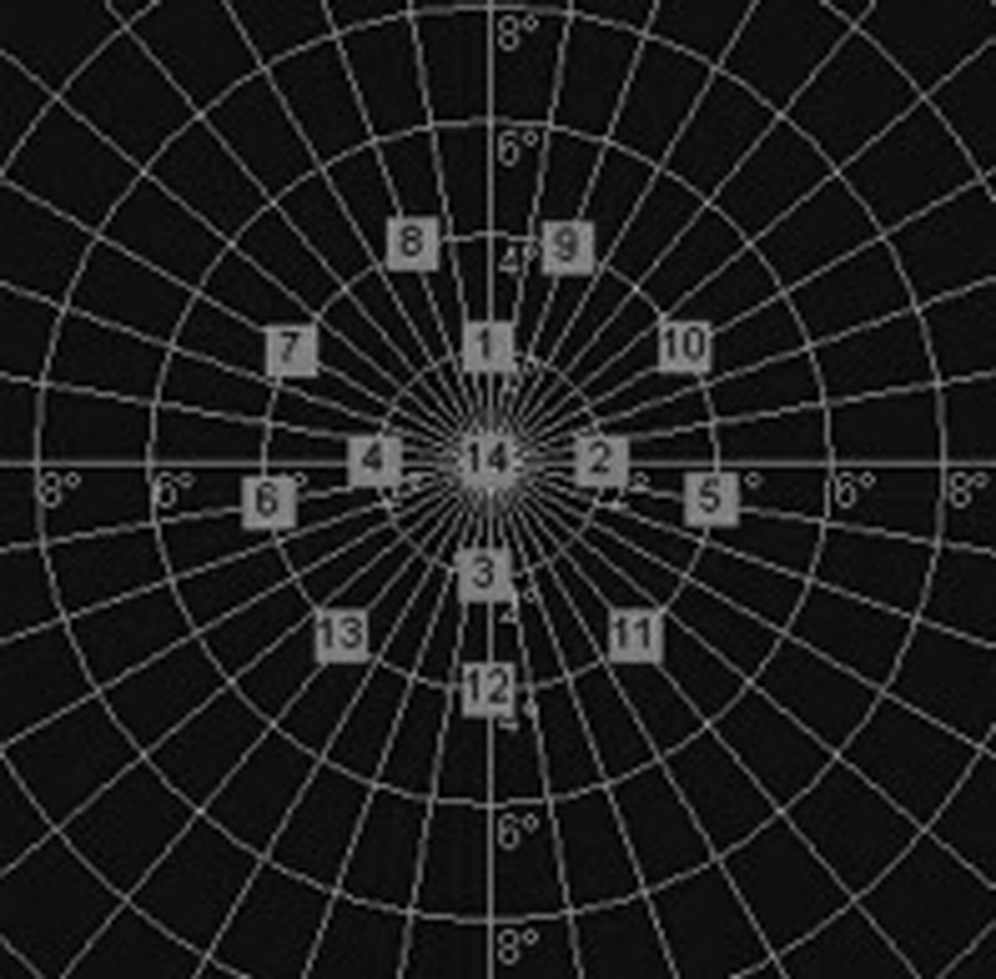
Location of the microperimeter points.

Fourteen locations centred on the fovea covering a circular area 8° in diameter were tested. The results of the fixation and microperimetry tests were displayed on color digital photographs acquired by the MP-1 colour camera. The mean retinal sensitivity was recorded as the mean of the 14 points while the mean right and left retinal sensitivity were calculated as the mean of 5 points to the right and left of the centre (Figure 1).

### Retinal thickness measurement

Central retinal thickness and macular volume (MV) were recorded directly from the computerised software of the Cirrus HD-OCT (Carl Zeiss Ltd, Welwyn Garden City, UK).

### Statistical methods

The data was entered into a database and analysed using SPSS. As the lower limit of normal of MRS is 105 words per minutes (wpm), we defined slow readers (SR) as those who read less than 105 words/min and normal readers (NR) read ≥105 letters. Student t-test was used to compare the variables between slow readers and normal readers.

The correlations as continuous variables between MRS and each recorded measure of vision (distance visual acuity, contrast sensitivity, reading acuity, CPS, fixation stability, log BCEA, right retinal sensitivity, left retinal sensitivity, mean macular sensitivity) were examined using Pearson's product moment correlation coefficient.

Stepwise multiple linear regression was performed to detect independent prognostic indicators for maximum reading speed. Then, a parsimonious model of maximum reading speed was created using the significant predictors. Statistical significance was set at p<0.05 for all analyses.

## Results

### Slow and normal readers

In total, 20 eyes of 20 patients (with 12 female and 8 male) were included in the study. The demographic, visual and macular morphological characteristics of the slow and normal readers are shown in table 1. Despite the fact that the distance visual acuity between the two groups were not significantly different, the fixation stability, contrast sensitivity and the mean retinal sensitivity along with the retinal sensitivity in the right side of the centre were significantly worse in the slow readers.

**Table 1:** SR  =  Slow reading group, NR  =  Normal reading group, SD  =  standard deviation, CST  =  Central Subfield Thickness, MV  = Macular Volume, BCVA  =  Best corrected visual acuity, CS  =  Contrast Sensitivity, RA  =  Reading acuity, CPS  =  Critical Print Size, MRS- Maximum reading speed BCEA  =  Bivariate Contour Ellipse Area, pt  =  point, MP  =  Microperimetry.

	SR (n = 10) Mean ± SD	NR (n = 10) Mean ± SD	
Age (years)	65.4±9.9	64.5 ±6.63	p = 0.81
Duration of diabetes (years)	18.4±4.9	16.9±5.3	p = 0.52
CST (microns)	278.7±79.0	263.7±49.16	p = 0.62
Average macular thickness (microns)	261.6±31.3	268.8±29.3	p = 0.602
MV (mm2)	10.1±0.58	8.9±3.02	p = 0.25
BCVA (LogMar)	76.7±6.91	78.8±4.05	p = 0.42
CS (letters)	30.6±3.31	35.1±1.37	p = 0.001*
RA	0.18±0.23	−0.01±0.295	p = 0.12
CPS	0.38±0.16	0.28±0.16	p = 0.18
Log BCEA	3.70±0.798	3.13±0.326	p = 0.013*
Central 14 pt MP value (dB)	11.31±3.38	14.79±3.09	p = 0.027*
MP value Right 5 pt subfield (dB)	10.4±4.98	14.8±3.33	p = 0.033*
MP value Left 5 pt subfield (dB)	12.6±2.80	14.5±3.15	p = 0.17

*denotes significant p<0.05.

### Correlation between maximum reading speed (MRS) and other parameters

The correlations as continuous variables between MRS and each recorded measure of vision (distance visual acuity, contrast sensitivity, reading acuity, CPS, fixation stability, log BCEA, right retinal sensitivity, left retinal sensitivity, mean macular sensitivity) were examined using Pearson's product moment correlation coefficient (Table 2).

**Table 2:** Correlation between maximum reading speed (MRS) and other parameters.

	MRS *R*	MRS *p-value*
Age (years)	0.045	0.426
Gender	−0.043	0.429
Duration of diabetes (years)	−0.191	0.419
Average macular thickness (microns)	0.153	0.518
Central sub-field thickness (microns)	−0.035	0.883
Macular volume (mm2)	−0.250	0.287
BCVA (letters)	0.253	0.282
Contrast sensitivity (letters)	0.729	**0.000**
Critical print size	−0.244	0.301
Reading acuity	−0.256	0.276
Log BCEA	−0.539	**0.007**
MP value Right 5 pt subfield (dB)	0.389	0.090
MP value Left 5 pt subfield (dB)	0.222	0.346
Central 14 pt MP value (dB)	0.413	0.070

BCEA  =  Bivariate Contour Ellipse Area, pt  =  point, MP  =  Microperimetry, BCVA  =  best corrected visual acuity.

### Regression models

The independent positive predictors of MRS were contrast sensitivity, log BCEA and macular sensitivity of the right parafoveal area (Table 2). The overall regression was significant, p = 0.001. The R^2^ was 0.707 and the age and sex adjusted R^2^ was 0.602, indicating that the set of predictors were able, as a group, to predict 70.7% of the variance in MRS in the sample. The adjusted R^2^ of 60.2% is the estimated amount as extrapolated to the population.

The beta coefficient of 8.091 for contrast sensitivity means that the group with good contrast sensitivity has a MRS of 8 letters higher than that of the group with poor contrast sensitivity. LogBCEA and macular sensitivity of the right parafoveal area also showed significant association with MRS but was no longer showing association when controlled for all other variables (Table 3).

**Table 3:** Multiple regression model showing significant predictors of MRS.

Model	Unstandardized Coefficients		Standardized Coefficients		
	B	Std. Error	Beta	t	Sig.
(Constant)	−42.400	79.142		−.536	.601
Age (years)	−.717	.628	−.200	−1.141	.273
Sex	13.130	10.367	.224	1.267	.226
CS (letters)	8.091	2.006	.927	4.034	.001
Log BCEA	−12.179	7.015	−.293	−1.736	.104
MP1right (dB)	−1.758	1.569	−.280	−1.120	.282

## Discussion

In this study, stable laser treated DME patients were defined as patients with relatively good vision and without laser treatment for over 12 months. They could have similar demographic data, duration of diabetes, OCT findings, BCVA, reading acuity and critical print size, and yet have significantly different reading speed. This study highlights the importance of including reading speed in the clinical assessment, as BCVA and OCT are probably the only routine tests carried out in this group of patients.

The similarity of reading acuity and critical print size in both groups were slightly surprising. However it was not totally unexpected. The reading acuity depends on the foveal function. If the foveal area is not affected, then the reading acuity is unlikely to be affected akin to distance visual acuity which also estimates foveal function only. This study explains why some patients who report difficulty with reading despite good distance and near acuity, may be slower readers. In other words, they could see the individual letters or words, but are not able to read quickly.

The slow reading group had poorer contrast sensitivity, reduced retinal sensitivity and less stable fixation. Most interestingly the reduced retinal sensitivity findings were driven by the microperimetry value on the right subfield (nasal to fovea in the right eye and temporal to fovea in the left eye). We speculated that this might explain the fixation instability when the patient is attempting to find the next word in the sentence.

All findings were not unexpected but have not been previously documented in this group of patients. Diabetic retinopathy and diabetic macular edema can reduce contrast sensitivity and these changes cannot be altered. Conventional laser treatment can reduce contrast sensitivity and retinal sensitivity further by causing collateral neuroretinal damage. Subthreshold micropulse laser, might be the preferred choice of laser if laser is needed [6] as it can improve retinal sensitivity after the reabsorption of the edema. It is unclear whether anti-VEGF treated patients would behave differently. However, as the latter is less likely to cause retinal damage, it is possible that the retinal sensitivity reduction would be less. A prospective randomised controlled trial, including reading speed, would be needed to clarify this issue.

The concept of a reading corridor was suggested a while ago. In languages based on reading from the left to right, the next word would project onto the retina nasal to the fovea in the right eye and temporal to the fovea in the left eye. A relative scotoma would make it harder for the patient to find the next word and hence reduce reading speed as observed in our study.

Fixation instability has been associated with reduced reading speed in patients with AMD [7]. Acuity declines when fixation instability is overcompensated, showing limited tolerance to increased retinal image motion suggesting fixation instability does not improve visual acuity and may be a consequence of poor oculomotor control. This overcompensation also leads to reduce reading speed in macular diseases as reported by Macedo and colleagues [7].

One of the major limiting factors of the study is the small number of study eyes, however, the entry criteria were tight and finding eyes which met all the criteria, and patients who were willing to be extensively studied was more difficult than expected. Furthermore, even with the relatively small number, the differences in contrast sensitivity and retinal sensitivity were highly significant statistically, and were not unexpected.

Another limiting factor is that all the tests were carried out monocularly while most people would normally read with both eyes. Kabanarou and colleagues have shown that the fixation loci can change from monocular to binocular viewing in AMD patients [8]. It is uncertain how that would affect our results and would warrant further evaluation.

Another limiting factor is that the contrast sensitivity decreases with increasing eccentricity [9] but the retinal sensitivity as measured in microperimetry does not vary more than 2 dB within the central +/−4 degrees other than the foveal sensitivity. Moreover, it is unclear how the weightings obtained by Baldwin and colleagues would apply to our clinical cohort, because they used healthy, psychophysically-experienced observers and stimuli very different to ours. Hence, we have applied statistical analysis to the whole central area and also the right and left subfield including equal numbers of microperimetry points at 2 and 4 degrees from fixation.

We were not able to co-localise the lasered area and the microperimetry points in this study, however when edema is located within the reading corridor, one might want to consider a treatment modality which is less likely to cause collateral damage in order to preserve reading speed. Further studies on the effect of such modalities such as subthershold laser and anti-VEGF agents on reading speed needs further evaluation.
